# PRR-Mediated Immune Response and Intestinal Flora Profile in Soybean Meal-Induced Enteritis of Pearl Gentian Groupers, *Epinephelus fuscoguttatus*♀ × *Epinephelus lanceolatus*♂

**DOI:** 10.3389/fimmu.2022.814479

**Published:** 2022-02-28

**Authors:** Wei Zhang, Beiping Tan, Junming Deng, Qihui Yang, Shuyan Chi, Aobo Pang, Yu Xin, Yu Liu, Haitao Zhang

**Affiliations:** ^1^ Laboratory of Aquatic Animal Nutrition and Feed, College of Fisheries, Guangdong Ocean University, Zhanjiang, China; ^2^ Aquatic Animals Precision Nutrition and High Efficiency Feed Engineering Research Center of Guangdong Province, Zhanjiang, China; ^3^ Key Laboratory of Aquatic, Livestock and Poultry Feed Science and Technology in South China, Ministry of Agriculture, Zhanjiang, China

**Keywords:** *Epinephelus fuscoguttatus♀* × *E. lanceolatus♂*, soybean meal, intestinal flora, pattern recognition receptors, enteritis

## Abstract

Pattern recognition receptors (PRRs) can recognize microbial-specific pathogen-associated molecular patterns, initiate signal cascade transduction, activate the expressions of host immunity and proinflammatory genes, and, ultimately, trigger an immune response against identified pathogens. The present study focused on two outcomes of feeding pearl gentian groupers with high levels of soybean meal (SBM): (1) growth performance and (2) the intestinal environment, including tissue structure, flora profile, and immune responses. Some 720 groupers were randomly divided into three groups (*n* = 4): (1) controls, fed a 50% fish meal feed (FM), (2) with 20% of the FM substituted with SBM (SBM20), and (3) 40% of the FM substituted with SBM (SBM40). The fish were fed these iso-nitrogenous and iso-lipidic diets for 10 weeks. They were kept in containers with 1 m^3^ of water under natural light and temperature levels. The experimental results demonstrate that the SBM diets significantly degraded growth performance and intestinal physiology. Typical enteritis characteristics and immune fluctuations appeared, as reflected by the enzyme activities of total superoxide dismutase and lysozyme, and the contents of immunoglobulin M, complement 3, and complement 4. 16SrDNA high-throughput sequencing showed that the intestinal flora was significantly affected, with the abundance of harmful bacteria, such as *Vibrio* and *Streptococcus*, increasing with dietary SBM level. Based on “3 + 2” full-length transcriptome sequencing, three triggered PRRs were found in the intestine: the RIG-like receptor, NOD-like receptor, and Toll-like receptor signaling pathways. The intestinal flora variations were significantly correlated with the activation of the three PRR signaling pathways by canonical correlation analysis. These culminated in the transcriptome activation of NF-κB, IRFs, and costimulatory molecules, ultimately promoting the expressions of proinflammatory cytokines, interferons (IFNs), chemokines, and other molecules vital to the innate and/or adaptive immune responses. This study provides new information for diagnosing and preventing SBMIE in aquaculture fish.

## Introduction

Fish meal (FM) is a useful source of protein and lipid for fish. Some 70% of the global FM supply is used in aquafeeds (1). With the continuous expansion of aquaculture, the demand for FM is increasing. However, the rising prices and declining production of FM have forced the aquaculture industry to find innovative alternatives (2).

An ideal and widely used alternative is soybean meal (SBM), which has a reliable supply, moderate price, and balanced amino acid composition. However, soybean meal contains a variety of antinutritional factors, such as saponins, soybean lectins, antigenic proteins, trypsin inhibitory factors, phytic acid, soybean oligosaccharides, and isoflavones (3, 4). Common soybean meal raw materials can only be added to feed at low proportions, as too high a proportion may alter the intestinal microbiome and cause enteritis in the distal intestine (DI), as has been reported in Atlantic salmon, rainbow trout, carp, and zebrafish (5–7). Our previous pilot experiment found that SBM substituted for FM at proportions of 20% and 40% (in a feed with 50% basal FM protein) induced obvious enteritis and immune responses in the DI tissue of pearl gentian groupers, and negatively affected their intestinal flora. During this experiment, we found that some pattern recognition receptors (PRRs) and their mediated immune-related NF-κB signaling pathways were activated, which may play a significant role in soybean meal-induced enteritis (SBMIE) in pearl gentian groupers. However, its exact mechanism needs further study.

In 1993, the American immunologist Janeway put forward the famous pattern recognition theory, which holds that some pathogens or their products share a specific molecular structure that is highly conserved in evolution (8). The highly conserved molecular structures are called pathogen-associated molecular patterns (PAMPs) and include lipopolysaccharide (LPS), peptidoglycan, cilia, lipoteichoic acid (LTA), bacterial DNA, and viral RNA/DNA (9, 10). PRRs are nonclonally distributed molecules that are mainly expressed on the surface of natural immune cells. They can sense one or more PAMPs that are shared among many microorganisms or endogenous damage-associated molecular patterns (DAMPs) to activate immune cells and mediate innate immune responses (11–14). In the vertebrate immune system, PRRs are mainly divided into five classifications according to the protein domain homology: (1) Toll-like receptors (TLRs), (2) nucleotide oligomerization domain (NOD)-like receptors (NLRs), (3) retinoic acid-inducible gene I (RIG-I)-like receptors (RLRs), (4) C-type lectin receptors (CLRs), and (5) absent in melanoma-2 (AIM2)-like receptors (ALRs) (15–17). Among these five types of PRRs, the fifth has not been found in teleosts (14, 18). All PRRs have a field that can recognize PAMPs and connect a domain that interacts with downstream signal molecules and, sometimes, there is an intermediate domain. Based on ligand (PAMPs) recognition, all of these PRRs rely on the interaction of specific linker proteins to initiate signal cascade transduction. Finally, the PRR signal activates particular transcription factors (NF-κB or IRFs) and kinases (MAPK) and generates a streaming network (19–21). Through this streaming network, the PRRs regulate a number of host immune and proinflammatory genes and coordinate the immune response to the identified pathogens (22). Therefore, PRRs only recognize microbial-specific PAMPs and do not recognize host structures (9, 23).

*Epinephelus fuscoguttatus*♀ × *E. lanceolatus*♂ is a regular marine predatory fish and is one of the most significant aquaculture fish in the Indo-Pacific region and elsewhere. It has tender meat, rapid growth, and strong disease resistance, giving it a high market value and broad market prospects (24). In this study, with the help of multiomics technology, we analyzed the roles of the three PRRs—RLRs, NLRs, and TLRs—and their mediated immune-related signal pathways, such as NF-κB, in the intestinal mucosa of pearl gentian groupers with SBMIE.

In recent years, the continuous discovery of PRRs and the in-depth exploration of immune mechanisms have provided novel pathways to finding more efficient and safe adjuvants for disease prevention. The results of this experiment provide a theoretical reference to solve the fish intestinal health issues caused by replacing dietary FM with plant protein.

## Materials and Methods

### Experimental Diets

The three iso-nitrogenous, including about 50% of crude protein and 10% total lipid of iso-lipidic. In the present study, the experiment set up the experimental diets, and the composition and chemical analysis of which are displayed in **Table 1**. Red FM contained 72.53% of crude protein and 8.82% of crude lipid, which was purchased from Corporación Pesquera Inca S.A.C., Bayovar Plant, Peru. The study also used SBM, which contained 48.92% of crude protein on dry matter basis and was supplied by Zhanjiang Haibao Feed (Zhanjiang, China). The SBM protein was used to replace proteins to form experimental diets. The one that was used to replace 0 FM protein was named FM (control), the one which was used to replace 20% FM protein was named SBM20, and the third one which was used to replace 40% FM protein was named SBM40. The researchers in this study added lysine and methionine to the experimental feed for the imbalance compensation. The researchers also grounded the ingredients into fine powder. After finishing grounding, they sieved it through 60-mesh size. The last step is to weigh accurately based on the formula. They used the sequential expansion method to mix the microconstituents homogeneously. For gaining a homogeneous mixture, the researcher then added and fully mixed the deionized water and the lipids. After gaining a dough, the researchers used the pelletizers, which were 2.0 and 3.0 mm diameter, for the passing through of the dough. Before they were used, the pellets were dried by air to 10% moisture and stored in plastic bags under the temperature of −20°C. What was demonstrated in **Supplementary Table S1** was the detection of essential amino acids in the diets. Based on the GB/T23788-2009 and GB/T16631-2008, which are the Chinese national standards, the contents of ANFs in SBM were detected. In addition, the study investigated Soyasaponin I by using HPLC-ESI/MS^2^. The results are shown in **Supplementary Table S2**.

**Table 1:** Formulation and proximate composition of the experimental diets (%, dry matter).

Ingredients (%)	Diets		
	FM	SBM20	SBM40
Red fish meal	50.00	40.00	30.00
Soybean meal	0.00	14.83	29.65
Vital wheat gluten	5.00	5.00	5.00
Wheat flour	18.00	18.00	18.00
Casein	4.60	4.60	4.60
Gelatin	1.00	1.00	1.00
Fish oil	3.02	3.75	4.48
Soybean oil	2.00	2.00	2.00
Soybean lecithin	2.00	2.00	2.00
Microcrystalline cellulose	11.48	5.74	0.00
Calcium monophosphate	1.50	1.50	1.50
Ascorbic acid	0.05	0.05	0.05
Choline chloride	0.50	0.50	0.50
Vitamin premix^a^	0.30	0.30	0.30
Mineral premix^b^	0.50	0.50	0.50
Ethoxyquin	0.05	0.05	0.05
Lysine^c^	0.00	0.12	0.24
Methionine^c^	0.00	0.06	0.13
Proximate composition) (%, dry matter)			
Crude protein	50.97	50.56	50.85
Crude lipid	10.15	10.50	10.44

a Vitamin premix consisted of the following (g/kg premix): VB_1_, 17.00 g; VB_2_, 16.67 g; VB_6_, 33.33 g; VB_12_, 0.07 g; VK, 3.33 g; VE, 66.00 g; retinyl acetate, 6.67 g; VD, 33.33 g; nicotinic acid, 67.33 g; d-calcium pantothenate, 40.67 g; biotin, 16.67 g; folic acid, 4.17 g; inositol, 102.04 g; cellulose, 592.72 g.

b Mineral premix consisted of the following (g/kg premix): FeSO_4_·H_2_O, 18.785 g; ZnSO_4_·H_2_O, 32.0991 g, MgSO_4_·H_2_O, 65.1927 g; CuSO_5_·5H_2_O, 11.0721 g; CoCl_2_·6H_2_O (10%), 5.5555 g; KIO_3_, 0.0213 g; KCl, 22.7411 g; Na_2_SeO_3_ (10%), 0.5555 g; zeolite powder, 843.9777 g.

c Lysine and methionine were added to balance amino acid with FM control group.

### Feeding Trial and Culture Condition

The healthy juvenile groupers, which originally weighed around 9 g, were bought from Zhanjiang, China. All fish that were fed by commercial diets, which were purchased from Haida Aquatic Feed (Zhanjiang, China), were adapting to experimental conditions for an entire week. Before the official experiment, all fish could not eat any food for 24 h. After that, the fish were firstly anaesthetized using eugenol and then were divided into three experimental groups. The fish with similar size were randomly placed into a 1,000-L cylindrical fiberglass tank. A total of 60 fish were contained in each tank. The fish was fed by experimental diets from 8:00 am to 4:00 pm twice per day with four replicates. The process lasted 10 weeks until the fish reached the level of apparent satiation. Each feeding started from the FM control group. Feed consumption was counted by our previous method (25). The experiment was carried out in the inner cultural system of the Marine Biological Research Base, Zhanjiang, China. Every container was then continuously aerated by using air stones. During the experiment, light cycle should be applied under natural conditions. The temperature should be controlled under 29°C ± 1°C. The ammonia and the nitrate were below 0.03 mg L^−1^. The dissolved oxygen involved in this study was no less than 7 mg L^−1^. Within the first 14 days, the researchers changed 60% of the water in each container daily, then nearly 100% water was changed daily.

### Sampling Collection

When the culture experiment finished, the fish have fasted for 24 h. Before the fish were sampled, the fish were anaesthetized with eugenol (1:10,000). The fish, which were contained in each container, were then calculated and weighted to count the results, including the weight gain rate (WGR), specific growth rate (SGR), feed conversion ratio (FCR), hepatosomatic index (HSI), and survival rate (SR). The next step was to randomly select six fish from each tank and collect the blood from the caudal vein and store it at 4°C overnight. After that, to obtain the serum, the blood was centrifuged at 3,500 rpm for 10 min. The blood was stored at −80°C to analyze the enzyme. Subsequently, fish abdomen was cut through the midline. The researchers gently pulled out the intestine so as to clear up the mesenteric artery and use deionized water to wash off the external residue. Some of the distal intestine (DI) and liver samples were immediately obtained and immersed into liquid nitrogen after placing into cryopreservation tubes and moved to −80°C to analyze the enzyme activities. The researchers cut a couple of parts from the DI samples into pieces and placed them into the tube containing RNAlater. After storing at 4°C overnight, the samples were transferred to −80°C for gene expression determination.


$$\text{Weight gain rate (WGR,\%)}=\frac{100\times (\text{final body weight}-\text{initial body weight})}{\text{initial body weight}}$$

$$\text{Specific growth rate (SGR,\%/day)}=\frac{100\times [\text{Ln }(\text{final body weight)}-\text{Ln (initial body weight})]}{\text{days}}$$

$$\text{Feed conversion ratio (FCR)}=\frac{\text{feed intake}}{\left(\text{final body weight}-\text{initial weight}\right)}$$

$$\text{Hepatosomatic index (HSI,\%)}=100\times \left(\frac{\text{hepatic weight}}{\text{body weight}}\right)$$

$$\text{Survival rate (SR,\%)}=100\times \left(\frac{\text{final fish number}}{\text{initial fish number}}\right)$$

For intestinal flora and transcriptome sequencing, from each container, eight fish were selected randomly. The DI tissues of the randomly picked fish were sampled and washed by using deionized water for removing the residual. The researchers placed those samples in cryopreservation tubes and immersed them in liquid nitrogen. Half of them were used for 16S high-throughput sequencing and the other half were used for transcriptome sequencing.

### Histological Observation of Enteritis

When the feeding trial finished, the researchers randomly selected three fish from each of the containers. The DI samples were assigned into two parts. Each of the two parts was placed in 4% paraformaldehyde universal tissue fixative (Servicebio Technology, Wuhan, China) for 24 h before further treatment (H&E and Tunnel staining) (26, 27). The histological evaluation of enteritis was based on the study of Zhang et al. (25).

### Physiological Indicators

In this study, the BCA assay method (Beyotime Biotechnology, Haimen, China) was applied to test the intestine, liver, and serum samples mentioned above. After that, fish ELISA kits were used to detect the enzyme activities of full superoxide dismutase (T-SOD) in DI tissues, lysozyme (LYS) levels in serum, as well as both alanine aminotransferase (ALT) and aspartate transaminase (AST) found in liver tissues. The fish ELISA kits were also used to detect the contents of IgM, complement 3 (C3), and complement 4 (C4) in DI. The ELISA kits used in this study were purchased from Shanghai Jianglai Biotechnology(Shanghai, China). More detailed steps of determination were included in the instructions.

The gene expressions of proinflammatory-related genes (*IL1β*, *IL12*, *IL17*, *IL32*, and *TNFα*) and anti-inflammatory-related genes (*IL5*, *IL10*, *TGFβ1*, *IgM*, *CD4*) in DI tissues were determined, and the primers of the genes were devised by Primer Premier 5.0 software (**Supplementary Table S3**). The internal reference gene is *β*-actin. All the detection methods were carried out according to the instructions. The template sequences of the primers were contained from the PacBio SMART full-length transcriptome sequencing database of pearl gentian grouper in our lab, and the raw reads of the PacBio SMRT and Illumina RNA-seq were deposited in the NCBI Sequence Read Archive with accession numbers PRJNA664623 and PRJNA664416, respectively. The *β*-actin is regarded as the internal control gene. Also, the study used real-time fluorescence quantitative PCR (RT-qPCR) (Mastercycler ep realplex, Eppendorf, Germany) instrument to examine the expressions of the above relevant genes. The PCR conditions are 95°C for 2 min with 1 cycle, 95°C for 15 s, 60°C for 10 s, and 72°C for 20 s with 40 cycles, respectively. The relevant levels of aimed genes were investigated using the 2^−ΔΔCT^ method (28).

### 16S High-Throughput Analysis of Intestinal Flora

Total RNA of pearl gentian grouper DI microflora was extracted by using E.Z.N.A.TM Kit (Omega Bio-Tek, Norcross, GA, USA) according to the manufacturer’s instructions. The sequencing detection and data analysis were performed by Gene Denovo Co., Ltd. (Guangzhou, China). The raw data were stored in the NCBI Sequential Read Archive (SRA) database under the accession number PRJNA666309. The detailed analysis steps are supplied in the **Supplementary Material**.

### Transcriptome Sequencing Analysis

The “3 + 2” full-length transcriptome sequencing was performed on the PacBio Sequel and Illumina HiSeq™ 4000 platforms. The analysis of sequencing and data were implemented by Gene Denovo Co., Ltd. (Guangzhou, China). The raw reads of “3 + 2” sequencing are placed in the NCBI SRA under accession numbers PRJNA664623 and PRJNA66441. The detailed procedures, which include the “3 + 2” transcriptome sequencing processes, are supplied in the **Supplementary Material**.

The gene expressions with |log2FC|>1 and *p* < 0.05 were identified as DEGs. The trend analysis of the DEGs in the FM, SBM20, and SBM40 groups was carried out in this study. The DEG genes with significant differences (*p* < 0.05) were annotated into the KEGG database, and the signal pathways relevant to the immune disease system and infectious diseases were significantly activated. Also, signal transductions were further analyzed (*p* < 0.05).

### Verification of the PRR-Related Pathways by RT-qPCR

To verify the accuracy of “3 + 2” full-length transcriptome sequencing data, 24 genes of PRR-mediated immune signaling pathways were selected for qRT-PCR, which include the following: *LGP2*, *MDA5*, *IPS-1*, *MITA*, *TRAF2*, *TRAF3*, *TRAF6*, *IKKγ*, *IKKβ*, *TAK1*, *IRF3*, *IRF7*, *IκBα*, *p65*, *JNK*, *P38*, *NOD1*, *NOD2*, *RIP2*, *MyD88*, *PI3K*, *AKT*, *IRAK4*, and *IRAK1*. The gene expressions of the different TLR types in this experiment are provided in **Supplementary Table S4**. The primer template source of all the genes were the same as those mentioned in *(Physiological Indicators)* (**Supplementary Table S5**). The internal control gene was *β-*actin. The gene expressions were detected by qRT-PCR (Mastercycler ep realplex, Eppendorf, Germany). The PCR reaction conditions and analysis method were the same as *(Physiological Indicators)*.

### Correlation Analysis

In order to understand the relationship between OTU abundance variations of intestinal flora and the expression of key genes in the three PRR signaling pathways and downstream inflammatory genes, the key DEGs in the PRR signaling pathways were analyzed by canonical correlation analysis (CCA) with OTU abundance with significant differences in internal flora and inflammatory genes in internal tissues. At the genus level, the flora with significant variations in OTU abundance includes *Photobacterium*, *Faecalibacterium*, *Stenotrophomonas*, *Vibrio*, *Neisseria*, *Bacteroides*, *Streptococcu*s, *unidentified*_*Rikenellaceae*, *Romboutsia*, and *Subdoligranulum*; the key genes analyzed in RIG-like receptor signaling pathway includes *LGP2*, *MDA5*, *IPS-1*, *TRAF2*, *TRAF3*, *TRAF6*, *TAK1*, *IRF3*, *IRF7*, *IκBα*, and *p65*; the key genes analyzed in NOD-like receptor signaling pathway includes *NOD1*, *NOD2*, *RIP2*, *TAK1*, *JNK*, *TRAF6*, *IκBα*, and *p65*; the key genes analyzed in TLR-like receptor signaling pathway includes *TLR5*, *TLR8*, *TLR9*, *TLR21*, *TLR22*, *MyD88*, *AKT*, *TAK1*, *IκBα*, *p65*, and *IRAK4*; and the inflammatory genes includes proinflammatory genes (*IL1β*, *IL12*, *IL17*, *IL32*, and *TNFα*) and anti-inflammatory-related genes (*IL5*, *IL10*, *TGFβ1*, *IgM*, *CD4*) in the intestinal tissues.

### Statistics

The CCA analysis was performed by the omicshare tools, a free online platform for data analysis (https://www.omicshare.com/tools). All the other data were analyzed using one-way ANOVA after the homogeneity variance test. The results were presented as mean ± SD, and the statistical analysis was conducted using SPSS 22.0 (SPSS Inc., Chicago, IL, USA). The *p* < 0.05 was accepted as statistically different.

## Results

### Growth Performance

**Table 2** shows that the WGR and SGR were lower in the experimental groups than in the FM control group (*p* < 0.05). On the contrary, FCR increased significantly (*p* > 0.05). There were no apparent differences between groups in terms of HSI and SR (*p* > 0.05).

**Table 2:** Effect of different levels of soybean meal protein substitute for fish meal protein on the growth of pearl gentian grouper (*n* = 4).

Parameters	FM	SBM20	SBM40
IBW (g)	12.55 ± 0.00	12.55 ± 0.01	12.55 ± 0.04
WGR (%)	485.14 ± 7.08^a^	464.36 ± 10.12^b^	426.50 ± 9.59^c^
SGR (%/day)	2.60 ± 0.02^a^	2.54 ± 0.03^b^	2.44 ± 0.03^c^
FCR	0.84 ± 0.01^a^	0.87 ± 0.02^b^	0.95 ± 0.02^c^
HSI (%)	2.43 ± 0.45	2.21 ± 0.36	2.18 ± 0.25
SR (%)	99.17 ± 0.96	99.58 ± 0.84	99.17 ± 0.96

The different letters represent significant differences.

### Histological Analysis

**Figure 1:**
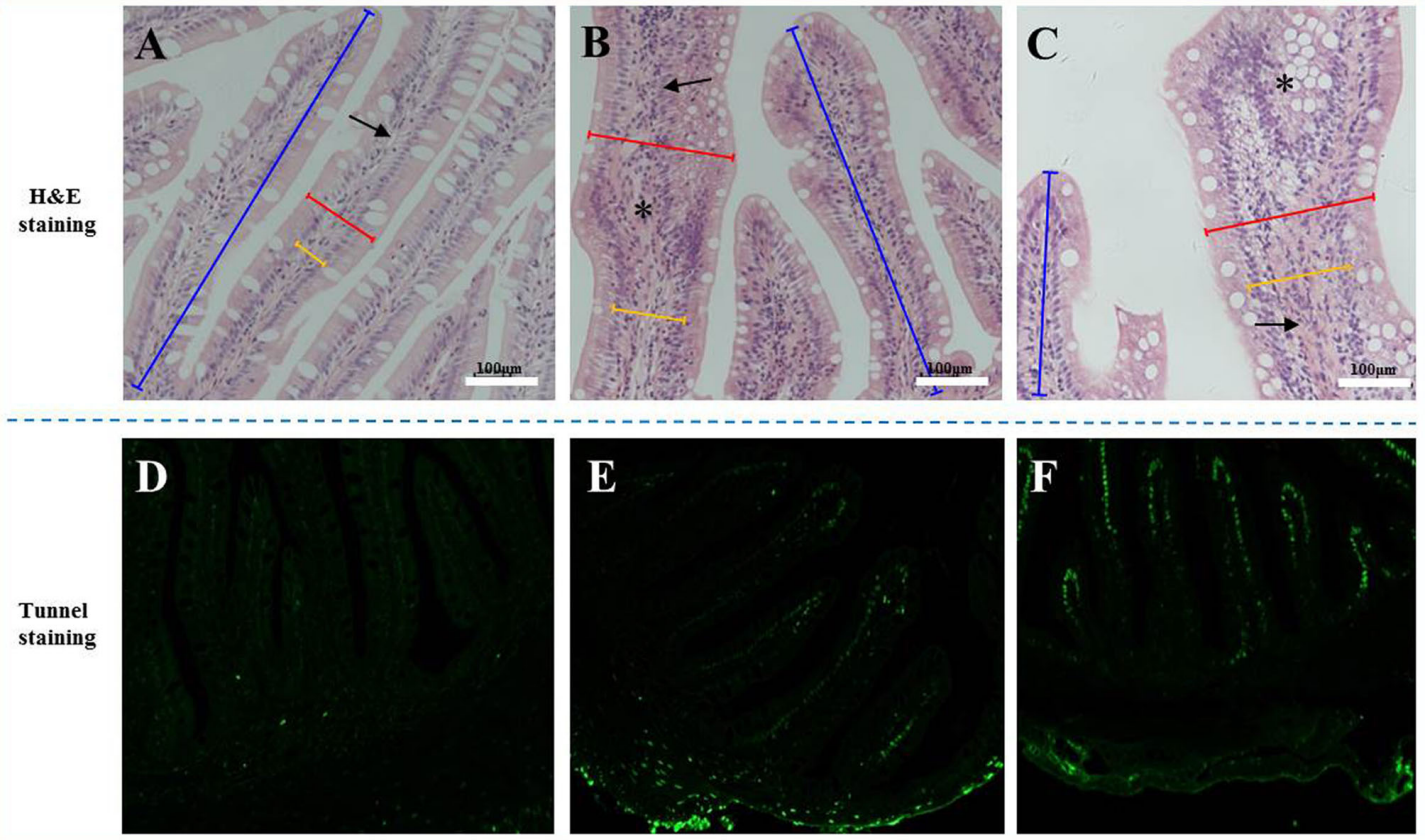
The tissue staining in the distal intestine sections of pearl gentian grouper. Representative histomorphological images from H&E **(A–C)** and Tunnel sections **(D–F)** of distal intestine of juvenile grouper on FM **(A, D)**, SBM20 **(B, E)**, and SBM40 **(C, F)** diets. **(A–C)** Representative images of increased width and cellular (leucocyte) infiltration (asterisk) of the lamina propria (arrows) in SBM20 and SBM40 groups. **(D–F)** The green fluorescence represented apoptosis cell; blue bar, the height of plica; red bar, the width of plica; and yellow bar, the width of lamina propria. FM, fish meal control group; SBM20, 20% SBM protein replacement level to FM protein; SBM40, 40% SBM protein replacement level to FM protein. Arrow-lamina propria; asterisk-inflammatory infiltration.

The H&E staining results in **Figure 1** show that the experimental substitution of FM with SBM caused DI enteritis. The SBM treatment resulted in a gradual increase in leucocyte infiltration and significantly affected the plica height/width, lamina propria width, and microvilli length (*p* < 0.05, **Table 3**). Tunnel staining also revealed that apoptotic cells gradually increased in number with increases in the SBM substitution level.

**Table 3:** The enteritis index in H&E sections of pearl gentian grouper distal intestine tissues (*n* = 10).

Parameters	FM	SBM20	SBM40
Plica height/width	7.80 ± 0.63^a^	5.31 ± 0.45^b^	3.31 ± 0.60^c^
Lamina propria width (μm)	16.65 ± 5.78^a^	41.78 ± 8.69^b^	63.53 ± 6.76^c^
Microvilli length (μm)	24.10 ± 2.06^a^	16.49 ± 1.94^b^	13.00 ± 1.50^c^

The different letters represent significant differences.

### Biochemical Index Determination

**Table 4** shows that the contents of IgM, C3, and C4 decreased significantly in DI tissues in groups SBM20 and SBM40 (*p* < 0.05), while the T-SOD and LYS enzyme activities significantly increased (*p* < 0.05). In addition, the ALT enzyme activity significantly increased in the liver tissues of group SBM40 (*p* < 0.05), while the AST enzyme activity significantly increased in groups SBM20 and SBM40 (*p* < 0.05).

**Table 4:** Effect of soybean meal on the enzyme activities of pearl gentian grouper (*n* = 4).

Parameters	FM	SBM20	SBM40
IgM (μg/mg)	94.33 ± 4.22^a^	79.47 ± 4.36^b^	64.70 ± 3.63^c^
T-SOD (μg/mg)	78.23 ± 9.95^a^	97.21 ± 10.53^b^	115.50 ± 12.07^c^
C3 (μg/mg)	85.58 ± 5.31^a^	73.31 ± 6.36^b^	61.70 ± 8.21^c^
C4 (μg/mg)	128.83 ± 10.17^a^	113.97 ± 11.04^b^	92.88 ± 5.62^c^
LYS (U/g)	5.45 ± 0.47a	7.06 ± 1.02^b^	7.95 ± 0.64^b^
ALT (U/g)	25.53 ± 3.34^a^	30.97 ± 3.84^a,b^	36.64 ± 4.13^b^
AST (U/g)	26.88 ± 4.02^a^	32.94 ± 4.25^b^	38.94 ± 4.34^b^

The enzyme activities of IgM, T-SOD, C3, and C4 were from distal intestine tissues; the enzyme activities of ALT and AST were from liver tissues; the enzyme activity of LYS was from serum. The different letters represent significant differences.

### Immune-Related Gene Expressions

**Figure 2:**
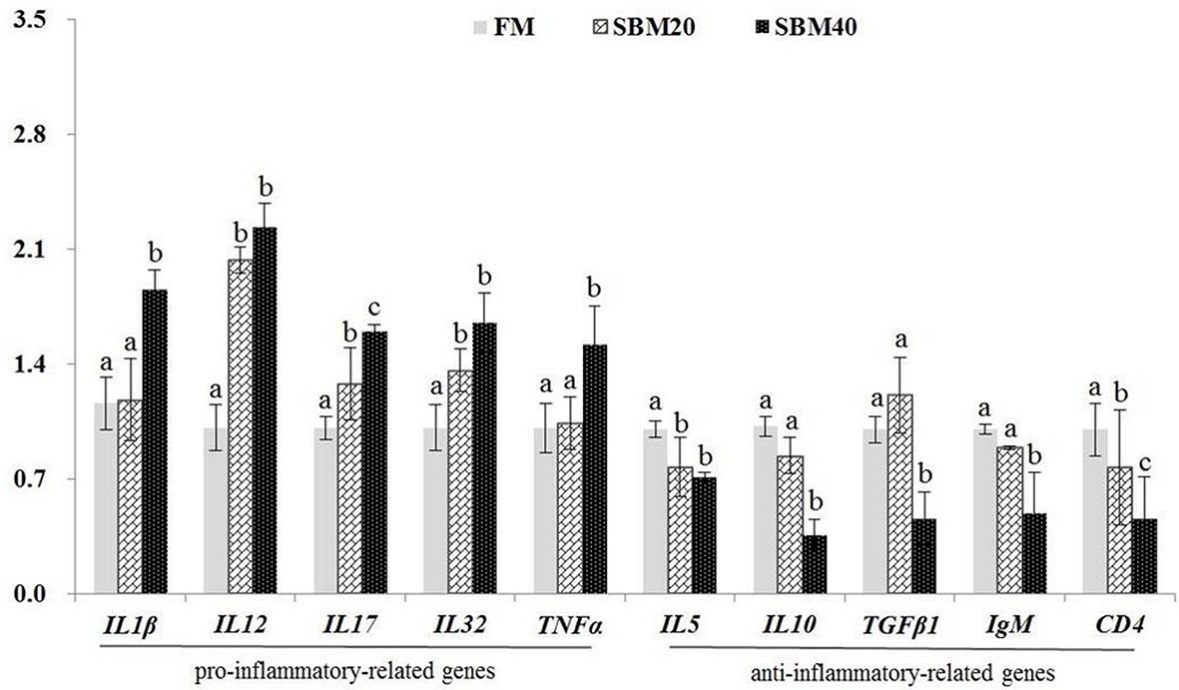
Effect of soybean meal substitution for fish meal on the immune-related gene expressions in the distal intestine tissues of pearl gentian grouper (*n* = 3). The different letters represent significant differences.

What was stated in **Figure 2** was that the expressions of proinflammatory genes *IL1β* and *TNFα* apparently increased in the SBM40 group (*p* < 0.05), and the expressions of the other genes *IL12*, *IL17*, and *IL32* significantly increased in the SBM20 and SBM40 groups (*p* < 0.05). The expressions of the anti-inflammatory-related genes *IL10*, *TGFβ1*, and *IgM* significantly decreased in the SBM40 group (*p* < 0.05), and the expressions of the other genes *CD4* and *IL5* significantly decreased in the SBM20 and SBM40 groups (*p* < 0.05).

### Sample Diversity Analysis of 16S Sequencing

**Figure 3:**
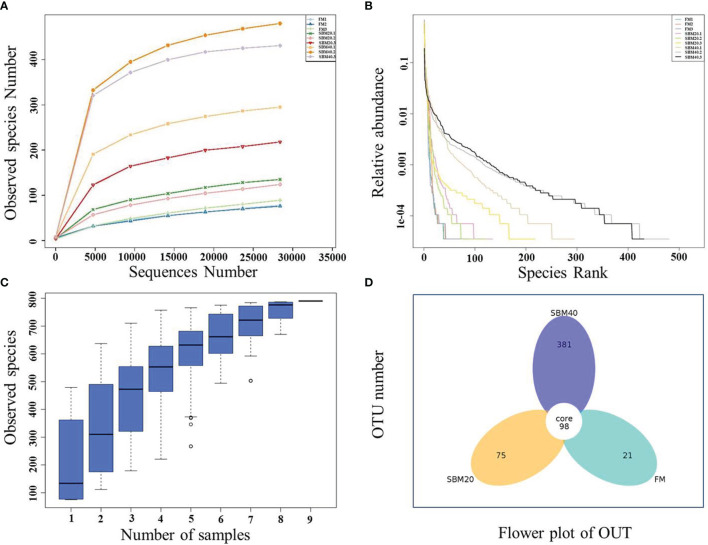
The complexity analysis of intestinal flora in soybean meal-induced enteritis of pearl gentian grouper (*n* = 3). **(A)** Rarefaction curve. **(B)** Rank abundance. **(C)** Species accumulation boxplot. **(D)** OTU flower plot.

**Figure 4:**
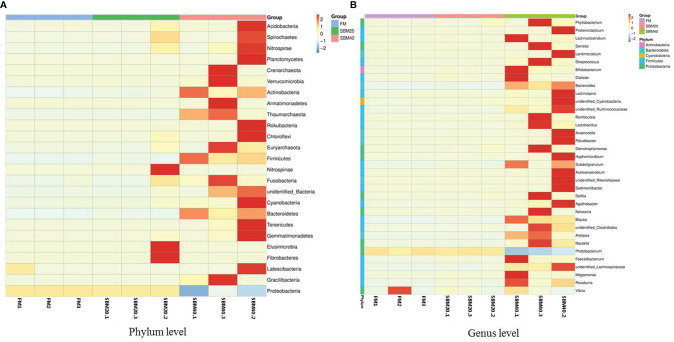
Cluster heatmap analysis of species abundance of intestinal flora in soybean meal-induced enteritis of pearl gentian grouper (*n* = 3). Vertical, the sample information; horizontal, the species annotation information. **(A)** The clustering heat map of OTU at plylum level; **(B)** The clustering heat map of OTU at genus level.

Sample diversity analysis of intestinal flora can demonstrate the abundance and diversity of the biological community based on a rarefaction curve, rank abundance, and species accumulation boxplot (*α* diversity). The results demonstrate that the rarefaction curve was plain, indicating that the number of sequencing data is reasonable (**Figure 3A**). Including more data would only identify a small number of new species (OTUs). The rank abundance curve reflects the richness and evenness of the species in each group of samples (**Figure 3B**). In addition, the species accumulation box gradually tended to be flat, indicating that the sample size was sufficient for data analysis (**Figure 3C**). **Figure 4** shows that different treatment groups could be clustered well according to the two levels of species and samples, indicating that the sample treatment was reasonable. The OTU number of intestinal flora in each group increased significantly with the dietary SBM addition amount (*p* < 0.05; **Figure 3D**). The significance test of differences among groups (*β* diversity) demonstrates that there were no apparent differences between groups FM and SBM20 (*p* > 0.05), but significant differences existed among other the groups (*p* < 0.05) (**Table 5**).

**Table 5:** The *β* diversity index differences of intestinal flora of pearl gentian grouper fed by different levels of soybean meal diets (*n* = 3).

Group-pair	Difference	p-value	Sig.
FM-SBM20	−1.66667	0.2488	
FM-SBM40	−5.33333	0.0065	^**^
SBM20-SBM40	−3.66667	0.0308	^*^

*Represents p < 0.05; **represents p < 0.01.

### Comparison of Intestinal Flora Composition and Abundance

**Figure 5:**
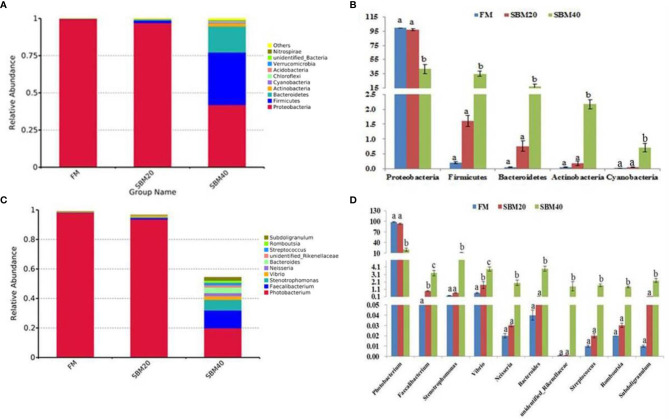
Comparison of bacterial composition and abundance of different soybean meal substitution levels [**(A, B)** phylum level; **(C, D)** genus level] (*n* = 3). The different letters represent significant differences.

The top 10 relatively abundant species were from the phyla Proteobacteria, Firmicutes, Bacteroidetes, Acidobacteria, Actinobacteria, Cyanobacteria, Chloroflexi, Acidobacteria, Verrucomicrobia, unidentified_Bacteria, and Nitrospirae. With increases in dietary SBM substitution level, the abundance of Proteobacteria decreased significantly (*p* < 0.05). The abundance of Firmicutes, Bacteroidetes, Actinobacteria, and Cyanobacteria significantly increased in the SBM40 compared with the control group (*p* < 0.05; **Figures 5A, B**; **Supplementary Table S6**).

The top 10 relatively abundant species were from the genera Photobacterium, Faecalibacterium, Stenotrophomonas, Vibrio, Neisseria, Bacteroides, Streptococcus, unidentified_Rikenellaceae, Romboutsia, and Subdoligranulum. Photobacterium was significantly less abundant in the SBM40 compared with the control group (p < 0.05). Faecalibacterium, Stenotrophomonas, Vibrio, Neisseria, Bacteroides, Streptococcus, unidentified_ Rikenellaceae, Romboutsia, and Subdoligranulum were significantly more abundant in the SBM40 (p < 0.05; **Figures 5C, D**; **Supplementary Table S7**).

### KEGG Enrichment Analysis of DEGs

**Figure 6:**
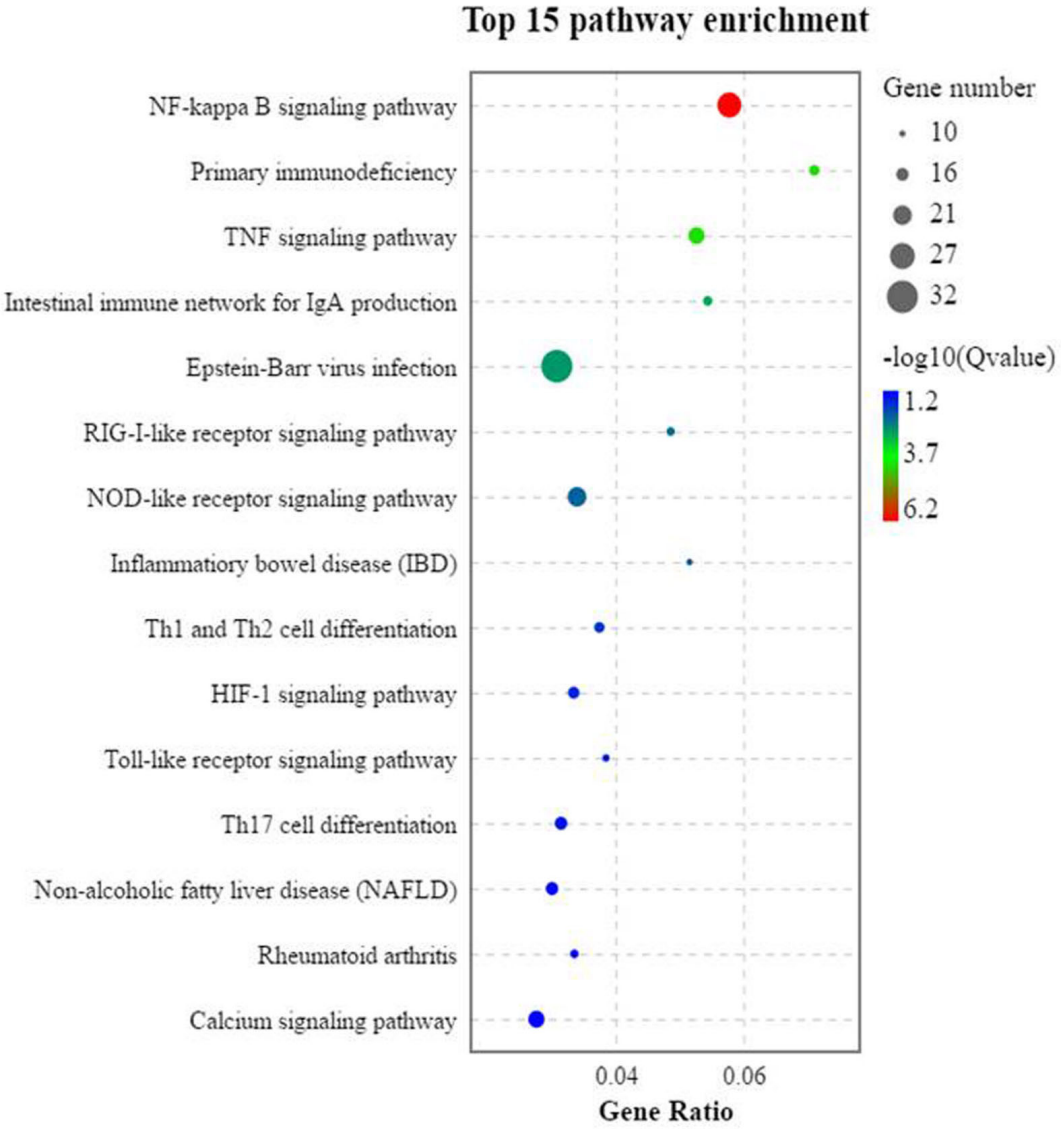
The trend analysis of the significantly activated KEGG pathways of the DEGs (*n* = 4).

The trend analysis of the DEGs showed that 1,296 genes had a significant upward trend (*p* < 0.05). KEGG trend enrichment analysis of the DEGs in all groups showed that a total of 266 pathways were enriched, 58 significantly (*p* < 0.05). Of all pathways, 78 are relevant to the immune diseases/system, infectious diseases, and signal transduction, of which 32 were greatly enriched (*p* < 0.05). This means that 55.17% (32/58) of the significantly enriched pathways are relevant to these three aspects (immune diseases/system, infectious diseases, and signal transduction). The majority of these important enrichment pathways are closely related to the development of immune responses, such as inflammation, which is often concurrent with immune response. All of the significant enrichment pathways are listed in **Supplementary Table S8**. Among these pathways, three pattern recognition receptors—the RIG-I-like receptor signaling pathway, NOD-like receptor signaling pathway, and Toll-like receptor signaling pathway—and their jointly mediated immune-related NF-κB signal pathway were significantly activated. These play important roles in SBMIE in pearl gentian groupers (**Figure 6**).

### Verification of the PRR-Related Pathways by RT-qPCR

**Figure 7:**
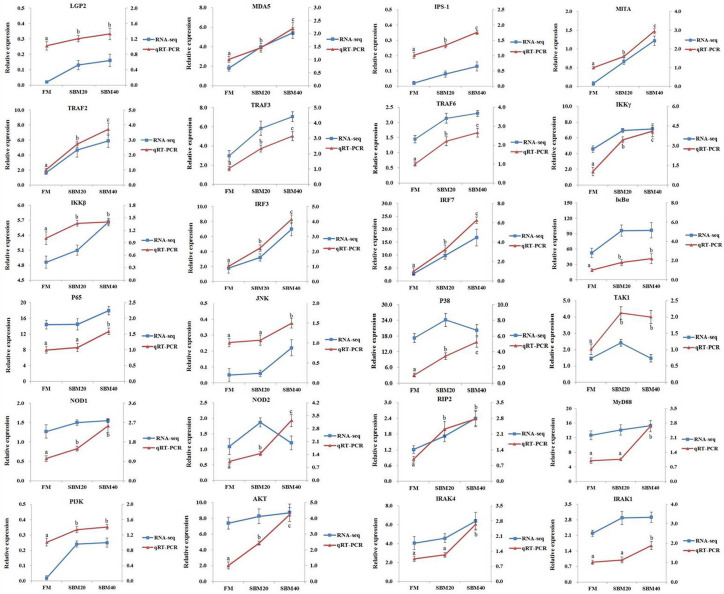
Comparison of transcriptome sequencing and RT-qPCR results (*n* = 4). The mRNA expression level of RT-qPCR was normalized by *β*-actin. The relative expression level in transcriptome sequencing was calculated by the FPKM value. The statistical results are expressed as mean ± SD. Different letters assigned to the lines represented significant differences between the groups at *p* < 0.05. FM, fish meal control group; SBM20, 20% SBM substitution level to FM; SBM40, 40% SBM substitution level to FM.

To verify the accuracy of the “3 + 2” full-length transcriptome sequencing data and the activation effects of the three PRR and NF-κB signaling pathways, 24 key genes were selected in these pathways for RT-qPCR. In general, the trends in the RT-qPCR results were consistent with those of transcriptome sequencing. This suggests that the transcriptome results are accurate (**Figure 7**) and further confirms the reliability of the “3 + 2” strategy.

**Figure 8:**
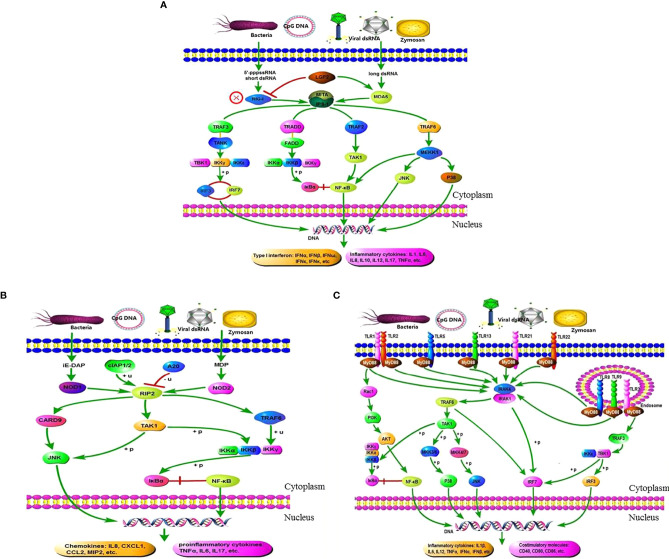
Exhibition of the three pattern recognition receptor signaling pathways and their mediated immunomodulatory effects in the DI tissues of SBMIE pearl gentian grouper. **(A)** There is no *RIG-I* gene in pearl gentian grouper, and the RIG-I like receptor signaling pathway was activated through MDA5 route; **(B)** there are several co-contained genes in the three PRR signaling pathway, indicating the interactions among them, and finally culminated in the transcriptome activation of NF-κB, IRFs, costimulatory molecules, etc., and finally promoted the expression of proinflammatory cytokines, interferons (IFNs), chemokines, and other molecules necessary for innate and/or adaptive immune responses. **(A)** RIG-like receptor signaling pathway. **(B)** NOD-like receptor signaling pathway. **(C)** Toll-like receptor signaling pathway.

**Figure 8** illustrates the activation effect of the three pattern recognition receptors on related signaling pathways after recognizing PRRs from pathogens or their products. We selected some typical genes in these pathways for analysis. When the pearl gentian grouper is in the SBMIE state, in the RIG-like receptor signaling pathway, the gene expressions of *LGP2*, *MDA5*, *IPS-1*, *MITA*, *TRAF2*, *TRAF3*, *TRAF6*, *IKKγ*, *IKKβ*, *TAK1*, *IRF3*, *IRF7*, *IκBα*, *p65*, *JNK*, and *P38* were verified to be significantly activated (*p* < 0.05), while the *RIG-I* gene has not been found in pearl gentian groupers. In the NOD-like receptor signaling pathway, the gene expressions of *NOD1*, *NOD2*, *RIP2*, *TAK1*, *JNK*, *TRAF6*, *IKKγ*, *IKKβ*, *IκBα*, and *p65* were verified to be significantly activated (*p* < 0.05). In the Toll-like receptor signaling pathway, the gene expressions of *MyD88*, *PI3K*, *AKT*, *IRAK4*, *IRAK1*, *TRAF6*, *TAK1*, *IKKβ*, *IκBα*, *p65*, *JNK*, *IRF3*, and *IRF7* were verified to be significantly activated (*p* < 0.05). Finally, the genes associated with immune regulation were significantly regulated (*p* < 0.05), some of which were evaluated above, such as *IL1β*, *IL12*, *IL17*, *IL32*, *TNFα*, *IL5*, *IL10*, *TGFβ1*, *IgM*, and *CD4*.

### Correlation Analysis

**Figure 9:**
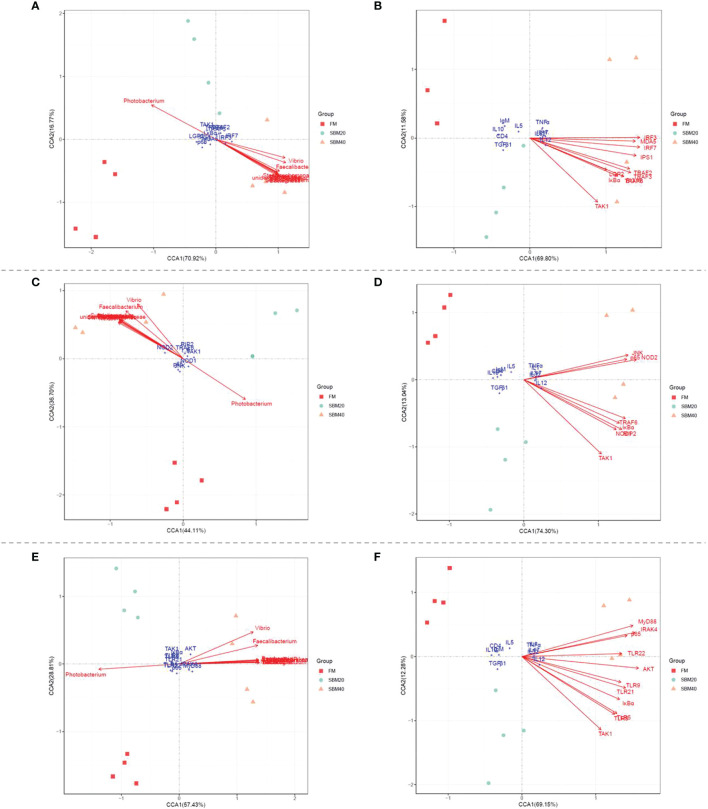
The canonical correlation analysis (CCA) between the OUT abundances of intestinal flora, the key genes in PRR signaling pathways, and the inflammatory genes (*n* = 4). **(A, C, E)** The CCA correlation analysis between intestinal flora and the key genes in RLR, NLR, and TLR signaling pathways, respectively. **(B, D, F)** The CCA correlation analysis between the key genes in RLR, NLR, and TLR signaling pathways and inflammatory genes, respectively. In CCA plot, the arrows represent explanatory variables and the points represent response variables. The lengths of the arrows represent the strength of the influence of the explanatory variable on the response variable; the longer lengths indicate greater influences. The angles between the arrows and coordinate axis represent the correlation between the explanatory variable and coordinate axis; smaller angles indicate stronger correlation. A sample point located in the same direction as the arrow indicates that the changes in explanatory and response variables are positively correlated, while in the opposite direction which indicates a negative correlation. The value in the coordinate axis label in the plot represents the interpretation proportion of the explanatory variable combination and the response variable variation.

**Figure 9** reveals the correlations between the OTU abundance of intestinal flora, key genes in the PRR signaling pathways, and inflammatory genes. Envfit tests were used to analyze whether the explanatory variables were significantly correlated with the response variables. The results showed that with the increase of SBM addition, the change of OTU abundance was significantly correlated with key genes in the three PRR signaling pathways (*p* < 0.05, **Supplementary Tables S9**–**S11**); except that *Photobacterium* was negatively correlated, the others were positively correlated. Similarly, in general, there was a significant correlation between the expression of key genes in the three PRR signaling pathways and the expression of downstream inflammatory genes (*p* < 0.05, **Supplementary Tables 12**–**14**); among them, it was positively correlated with proinflammatory genes and negatively correlated with anti-inflammatory genes.

## Discussion

The present study shows that SBM substitution caused vital negative influences on the growth performance of grouper fish, with changes in intestinal tissue structure, inflammatory characterization, and intestinal flora composition and abundance. It also triggered a variety of pathogen pattern-recognition receptor signal pathways and their mediated immune-regulatory responses. Our unpublished laboratory results indicate that the optimal SBM substitution level for pearl gentian groupers (initial weight, 17.01 ± 0.01 g) is 12.05% (in a 50% basal FM feed). An et al. (29) also found that the SBM substitution level (in a 65% basal FM feed) for *Epinephelus coioides* (initial weight, 84 ± 2.5 g) should not exceed 20%.

SBMIE is a common model used to study fish enteritis. It considers four main aspects: decline in mucosal fold height, swelling of the lamina propria and submucosa, loss of absorption vacuoles in normal intestinal nuclei, and serious infiltration of various inflammatory cells (30). Research indicates that the degree of severity of intestinal histopathological variations in fish SBMIE depends on the soy protein content and inclusion body levels (31). Histological analysis in the present study obtained similar results, which are also reflected in tissue apoptosis and the enteritis index.

Inflammation and immune responses are often concurrent (32, 33). The present study focused on the changes in intestinal immune response in pearl gentian groupers in the SBMIE state. The immune status of fish depends largely on humoral immunity and cellular immunity. In fish, humoral immunity includes nonspecific immunity and specific immunity (34). We first analyzed the overall immune status of groupers through immune-related indicators. Complement and LYS are significant components of nonspecific immunity, while IgM is a significant component of specific humoral immunity (35). Both C3 and C4 are crucial parts of complement system activation, with complement being an important component of microbial infection resistance (36). This experiment showed that dietary SBM addition reduced the contents of IgM, C3, and C4 in fish DI tissues, which indicates that SBM damages the immune functions of grouper fish. Gu et al. (37) reported that adding glutamine and arginine to a diet containing SBM increased the levels of C3, C4, and IgM in turbot serum and increased the activity of LYS. In addition, T-SOD is closely related to the immune status of the body; it plays a significant role in enhancing the defense function of macrophages and the immune function of the whole body and is often used as an indicator of nonspecific immune function (38). The changes in the above indexes observed in the experiments demonstrate that high substitution levels of SBM caused variations in the immune homeostasis of the groupers, which was also reflected in the LYS enzyme activity. Other studies also indicate that LYS is an important index of nonspecific immune function, which is a kind of hydrolase that acts on the cell walls of microorganisms; it can destroy the cell walls of bacteria, causing their dissolution and necrosis (39). Indeed, the intestinal flora also changed dramatically with increases in the SBM proportion in the experimental diets.

Healthy intestinal flora is crucial to fish health (40) and is influenced by many factors, including breeding cultivar, growth stage, and environmental factors. Obviously, the dietary composition is also crucial (41). Previous studies in our laboratory have demonstrated that the major bacteria in the intestinal flora of pearl gentian groupers are Proteobacteria, Bacteroidetes, Firmicutes, and Actinobacteria (42), and the present study obtained similar results. With increases in SBM addition, the abundance of Proteobacteria decreased but those of Firmicutes and Bacteroidetes gradually increased. Hu et al. (43) reported that adding 55% SBM to the diet of turbot significantly increased the abundance of intestinal Firmicutes and Bacteroidetes, which is consistent with our experimental results. The reason may be that Firmicutes and Bacteroidetes can use the unabsorbable oligosaccharides, phytoestrogens, and nonstarch polysaccharides in SBM as energy sources to proliferate massively (44). Meanwhile, some studies have stated that the occurrence of enteritis may occur alongside a decrease in Proteobacteria abundance and increases in Firmicutes and Bacteroidetes abundance (43). Vibronaceae is the main intestinal colonizing bacteria in marine fish. Many of the main flora are conditional pathogens, such as *Vibrio harveyi* and *Vibrio parahaemolyticus*, which can cause a disease in aquaculture animals such as fish and shrimp (45). Both *Photobacterium* and *Vibrio* pertain to the Vibrionaceae family. Hartviksen et al. (46) found that when Atlantic salmon were fed with 20% soybean protein concentrate, the relative abundances of the Vibrionaceae and Streptococcaceae families increased significantly in the intestine. *Streptococcus* is also a conditional pathogen; when the body’s resistance decreases, it can cause intestinal suppurative enteritis (47). The present study demonstrates that the abundance of *Vibrio* and *Streptococcus* increased following SBM addition to the diet. The abundance of *Photobacterium* decreased after dietary SBM addition in our experiments. Related research on *Litopenaeus vannamei* pointed out that *Photobacterium* may damage the intestinal morphology and immune mechanism (48). *Photobacterium* is a facultative anaerobic bacterium that is widely distributed in seawater and on the body surfaces and in the digestive tracts of some marine fish (49). Although the *Photobacterium* abundance was significantly lower in the SBM group, it was not clear whether this was caused by the decrease in the FM content of the SBM-substituted diets. Previous studies indicated that under pathological conditions, the variations in intestinal flora OTU abundance at the phylum or genus levels in mammals, including humans is sometimes opposite to that in fish (50–52). Similar results were found in this study. In humans, decreases in Bacteroidetes (53) and *Bacteroides* (54) levels are found in inflammatory bowel disease (IBD) patients. In contrast, several specific species of intestinal bacteria may have protective effects against IBD (55). For instance, species of *Bifidobacterium*, *Lactobacillus*, and *Faecalibacterium* may protect the host from mucosal inflammation (56, 57). In our previous research, species of these genera plus *Bacteroides* were also found in grouper intestines (42, 58). However, in the present paper, the abundances of *Faecalibacterium* and *Bacteroides* were significantly higher in the SBMIE pearl gentian grouper. The difference between this study and others may be mainly due to the different research objects. We will further analyze and verify the specific reasons for such differences in future experiments.

The intestinal flora plays a significant part in regulating its host’s intestinal mucosal immune response. The immunomodulatory effect of intestinal flora has been confirmed in the pathogenesis of IBD in humans and other animals (59). For example, patients with Crohn’s disease who were treated with a sterile ultrafiltrate of small intestinal exudate did not exhibit enteritis, while the reintroduction of small intestinal exudate caused enteritis (60). Pathogenic microbial infection can induce an inflammatory response in the host, which senses pathogenic signals and initiates a response *via* PRRs (61). In this experiment, the activation effects of multiple PRRs (RLRs, NLRs, and TLRs) were found in SBMIE groupers. In addition, previous analyses of plant protein-induced fish enteritis have found conservative changes in some signaling pathways, such as NF-κB (62), which is consistent with the present study. Therefore, we focused on the analysis of the three PRRs (TLRs, NLRs, and RLRs) and their comediated immune-related NF-κB signaling pathways.

In general, RLRs belong to intracellular PRRs, which can recognize viral RNA in the cytoplasm, induce the production of interferon (IFN) and proinflammatory cytokines through RLR cascade signaling, and play a very important role in the establishment of natural antiviral immunity (63). The family members of RLRs mainly include RIG-I, MDA5, and LGP2. LGP2 is homologous to RIG-I and MDA5 but lacks the helicase of CARD. LGP2 contains a repressor domain, which may be the control switch of innate immunity (64). RLRs are expressed in various virus-infected cells and directly recognize and perceive the viral components entering the cytoplasm. During viral infection, a large number of double-stranded RNA (dsRNA) are produced in cells. After recognizing the dsRNA by RIG-I and MDA5, the NF-κB and IRF-3/7 signaling pathways cause the production of type I interferon, which has an antiviral effect (65). However, in this experiment, the RIG-I gene was not found in the full-length transcriptome sequence of the groupers, nor is it present in the published genome of its parent grouper, *Epinephelus lanceolatus* (NCBI accession number PRJNA516312). Currently, gene homologs of RIG-I, MDA5, and LGP2 have been found in teleost fish. Interestingly, RIG-I only exists in some bony fish genomes, such as those of carp and salmon, suggesting that the homologous gene has been lost from some specific fish genomes or has differentiated into an unknown new gene (66). However, MDA5 and LGP2 are ubiquitous in all teleost genomes (67). Therefore, we speculate that in SBMIE pearl gentian groupers, the RIG-I receptor signaling pathway mainly mediates and activates the downstream immunomodulatory effect *via* the MDA5 pathway.

NLRs are also intracellular PRRs and can detect PAMPs and some endogenous molecules in the cytoplasm and play a significant role in the innate immune system (68). At present, there are at least 23 studies on mammalian NLRs, of which NOD1 and NOD2 are the most studied and have been confirmed as related to IBD (69). Until recently, NLRs have been confirmed in different teleosts, such as zebrafish, Japanese flounder, and grass carp (70–72). In mammals, NOD1 senses the presence of bacterial pathogens through the recognition of PGN molecules that obtain meso-diaminopimelic acid. Mammalian NOD2 detects muramyl dipeptide (MDP) in both G^+^ and G^−^ bacterial PGN, and both receptors trigger an immune response *via* the activation of NF-κB (73). Signaling transduction of NLRs in teleosts has received little study (11). The present study found that, in SBMIE groupers, intracellular PAMPs such as MDP and iE-DAP are recognized by NOD1and NOD2, respectively. Then, NOD1 and NOD2 recruit the RIP2 adaptor, resulting in JNK and NF-κB activation and the subsequent transcription of inflammatory cytokines.

As type I transmembrane proteins, TLRs consist of an extracellular segment containing a leucine-enriched repeat region, a transmembrane region, and a cytoplasmic segment with a Toll/IL-1 receptor region. TLRs are the first and best-characterized innate immune receptors (74). So far, at least 20 TLR types have been found in various fish species (75). On the basis of full-length transcriptome sequencing, nine TLRs have been found in pearl gentian groupers, which are *TLR1*, *TLR2*, *TLR3*, *TLR5*, *TLR8*, *TLR9*, *TLR13*, *TLR21*, and *TLR22* (**Supplementary Table S3**). Except for *TLR8*, the other eight TLRs have been reported in *Epinephelus coioides* (58). Also, in addition to *TLR8*, the other TLRs found in this experiment have been reported as bacterial ligands in teleosts (11, 73). In mammalians, *TLR1*, *TLR2*, *TLR4*, *TLR5*, *TLR6*, and *TLR10* are expressed in the plasma membrane, whereas *TLR3*, *TLR7*, *TLR8*, and *TLR9* are localized within intracellular vesicles (76). Combined with our previous results, when TLRs (mainly *TLR5*, *TLR8*, *TLR9*, *TLR21*, and *TLR22*) are activated, the relevant adaptor proteins are reformed in the cytoplasm to trigger different signal cascades. In this experiment, the *TLR4* gene was not found in grouper fish and the expression of *TLR3* had no significant change; however, *MyD88* and the key genes of the NF-κB signaling pathway (*IκBα* and *p65*) were significantly increased with dietary SBM addition. This indicates that the TLR-MyD88-NF-κB signaling pathway is a crucial aspect in the immune regulation of SBMIE pearl gentian groupers.

In summary, this study revealed that a high substitution level of SBM has a significant negative impact on the growth performance of pearl gentian groupers and significantly influences their intestinal flora. Finally, PAMPs trigger multiple PRR signaling pathways that culminate in the transcriptome activation of NF-κB, IRFs, and costimulatory molecules. This can promote the gene expressions of proinflammatory cytokines, IFNs, chemokines, and other molecules necessary for innate and/or adaptive immune responses. The regulation of fish SBMIE is a complex, multifaceted issue. Further clarification of immune regulation and related PRR mechanisms is needed to provide further information on the diagnosis and prevention of fish SBMIE.

## Data Availability Statement

The datasets presented in this study can be found in online repositories. The names of the repository/repositories and accession number(s) can be found in the article/**Supplementary Material**.

## Ethics Statement

The animal study and all experimental methods were reviewed and approved by the ethics review board of Guangdong Ocean University. All of the procedures were performed in accordance with the relevant guidelines and regulations.

## Author Contributions

WZ took part in the whole process of the experiment and wrote the draft of this manuscript. BT designed and co-conceived the experiment. JD and QY participated in the experiments and revised the draft critically for important intellectual content. SC, AP, and YX revised the first draft. YL and HZ analyzed the data and approved the final version.

## Funding

This research was supported by the National Key R&D Program of China (2019YFD0900200), the National Natural Science Foundation of China (NSFC 31772864), and the China Agriculture Research System of MOF and MARA (CARS-47).

## Conflict of Interest

The authors declare that the research was conducted in the absence of any commercial or financial relationships that could be construed as a potential conflict of interest.

## Publisher’s Note

All claims expressed in this article are solely those of the authors and do not necessarily represent those of their affiliated organizations, or those of the publisher, the editors and the reviewers. Any product that may be evaluated in this article, or claim that may be made by its manufacturer, is not guaranteed or endorsed by the publisher.
