# Supplementary material for: PRR-Mediated Immune Response and Intestinal Flora Profile in Soybean Meal-Induced Enteritis of Pearl Gentian Groupers, Epinephelus fuscoguttatus♀ × Epinephelus lanceolatus♂
Source: Front Immunol. 2022 Feb 28;13:814479. doi: 10.3389/fimmu.2022.814479 (PMC8919722; doi:10.3389/fimmu.2022.814479)
Supplement: Supplementary file 1 [file Table_1.docx]

**Supplementary Table 1** The content of 17 amino acids in diets (%, dry matter)

| Amino acids | Diets | | |
| --- | --- | --- | --- |
|  | FM | SBM20 | SBM40 |
| Lysine | 3.02 | 2.93 | 2.88 |
| Methionine | 1.01 | 0.95 | 0.86 |
| Arginine | 2.53 | 2.43 | 2.47 |
| Threonine | 1.78 | 1.75 | 1.73 |
| Isoleucine | 1.86 | 1.78 | 1.82 |
| Histidine | 1.28 | 1.27 | 1.20 |
| Valine | 2.10 | 2.00 | 1.98 |
| Leucine | 3.28 | 3.16 | 3.20 |
| Phenylalanine | 1.94 | 1.88 | 1.96 |
| Tyrosine | 1.47 | 1.38 | 1.43 |
| Aspartate | 3.86 | 3.78 | 3.91 |
| Serine | 1.89 | 1.87 | 1.97 |
| Glutamate | 8.17 | 7.83 | 8.33 |
| Glycine | 2.48 | 2.42 | 2.29 |
| Alanine | 2.37 | 2.33 | 2.20 |
| Proline | 2.78 | 2.61 | 2.82 |
| Cystine | 0.49 | 0.49 | 0.51 |
