# Supplementary material for: PRR-Mediated Immune Response and Intestinal Flora Profile in Soybean Meal-Induced Enteritis of Pearl Gentian Groupers, Epinephelus fuscoguttatus♀ × Epinephelus lanceolatus♂
Source: Front Immunol. 2022 Feb 28;13:814479. doi: 10.3389/fimmu.2022.814479 (PMC8919722; doi:10.3389/fimmu.2022.814479)
Supplement: Supplementary file 2 [file Table_2.docx]

**Supplementary Table 2** Determination of anti-nutritional factors in SBM diets（mg/kg）

| Group | FM | SBM20 | SBM40 |
| --- | --- | --- | --- |
| Glycitin | 0 | 41.09 | 46.13 |
| Genistin | 0 | 59.46 | 93.19 |
| Glycitein | 0 | 0 | 26.17 |
| Genistein | 0 | 49.06 | 82.36 |
| Soyasaponin I | 0 | 2121.21 | 3082.41 |
| Daidzein | 0 | 162.26 | 302.79 |
| Soybean 7S globulin | 0 | 32421.06 | 42586.41 |
| Soybean 11S globulin | 0 | 33556.66 | 48911.94 |
