# Supplementary material for: PRR-Mediated Immune Response and Intestinal Flora Profile in Soybean Meal-Induced Enteritis of Pearl Gentian Groupers, Epinephelus fuscoguttatus♀ × Epinephelus lanceolatus♂
Source: Front Immunol. 2022 Feb 28;13:814479. doi: 10.3389/fimmu.2022.814479 (PMC8919722; doi:10.3389/fimmu.2022.814479)
Supplement: Supplementary file 3 [file Table_3.docx]

**Supplementary Table 3** The PCR primers for intestinal immune-related genes of pearl gentian grouper

| Gene | Forward5’-3’ | Revise3’-5’ | Size (bp) |
| --- | --- | --- | --- |
| *IL1β* | AAGGTGGACGCCAACAGACA | GTTCACTGCAGGCTCAGGGA | 153 |
| *IL12* | GACGGAGCATTTCCTGGTGG | TGCTCCAAGAGCTCGGGTAA | 172 |
| *IL17* | GAGAGGACGGTGTCTGTGTGG | CATGCACAGTTGAGGGTGTGG | 101 |
| *IL32* | CAGCAACAGTAGCAGCAGGC | CCATCCTCCTCAGCTCTGCC | 176 |
| *TNFα* | AACTGTGTGTCCCCACTGCC | CCACAGATGGCCCAGGTCAT | 81 |
| *IL5* | GGCCAACAGTCAAGATGTCTGCC | GAATGACCAGGAGCAGTTCAGTGT | 160 |
| *IL10* | ACACAGCGCTGCTAGACGAG | TAGACTTGTGCCACGACGGG | 142 |
| *TGFβ1* | CTTCTCCTCCTCCTCGCTGC | GATGTTGCTGAGGGCTTCGC | 195 |
| *IgM* | TGTCAATGACCCACTGAGCCTCG | TCCACTGCAAACTGCTGGGC | 105 |
| *cd4* | GGCGAGTGGACATGTGTTGC | CCCCAGGAGATGTGAGCAGG | 161 |
| *β-actin* | GGCTACTCCTTCACCACCACA | TCTCCAAGGCAACGGGTCT | 188 |
