# Supplementary material for: PRR-Mediated Immune Response and Intestinal Flora Profile in Soybean Meal-Induced Enteritis of Pearl Gentian Groupers, Epinephelus fuscoguttatus♀ × Epinephelus lanceolatus♂
Source: Front Immunol. 2022 Feb 28;13:814479. doi: 10.3389/fimmu.2022.814479 (PMC8919722; doi:10.3389/fimmu.2022.814479)
Supplement: Supplementary file 4 [file Table_4.docx]

**Supplementary Table 4** The gene expressions of different TLR types detected by RT-qPCR in distal intestinal tissues of SBMIE pearl gentian grouper (n=3)

| TLR type | FM | SBM20 | SBM40 |
| --- | --- | --- | --- |
| TLR1 | 1.02±0.17 | 1.04±0.11 | 1.16±0.18 |
| TLR2 | 1.00±0.13 | 1.11±0.06 | 1.22±0.11 |
| TLR3 | 1.01±0.16 | 1.06±0.14 | 1.19±0.13 |
| TLR5 | 1.00±0.06^a^ | 1.60±0.07^b^ | 1.65±0.06^b^ |
| TLR8 | 1.00±0.08^a^ | 1.72±0.09^b^ | 1.71±0.10^b^ |
| TLR9 | 1.01±0.08^a^ | 1.22±0.11^b^ | 1.35±0.08^b^ |
| TLR13 | 1.00±0.05 | 1.14±0.09 | 1.17±0.14 |
| TLR21 | 1.01±0.14^a^ | 1.47±0.13^b^ | 1.69±0.11^b^ |
| TLR22 | 1.00±0.11^a^ | 1.07±0.10^a^ | 1.35±0.14^b^ |
