# Supplementary material for: PRR-Mediated Immune Response and Intestinal Flora Profile in Soybean Meal-Induced Enteritis of Pearl Gentian Groupers, Epinephelus fuscoguttatus♀ × Epinephelus lanceolatus♂
Source: Front Immunol. 2022 Feb 28;13:814479. doi: 10.3389/fimmu.2022.814479 (PMC8919722; doi:10.3389/fimmu.2022.814479)
Supplement: Supplementary file 5 [file Table_5.docx]

**Supplementary Table 5** The PCR primers of the key genes in three PRRs signaling pathways of pearl gentian grouper intestinal tissues

| Gene | Forward 5'-3' | Reverse 3'-5' | Size (bp) |
| --- | --- | --- | --- |
| *LGP2* | AACTTTCCGTTGTAGCA | TATTCAGTGCGAGGGAT | 134 |
| *MDA5* | TTGGGTAAATTGTGCTT | TCTACTGGGATGTCCTTC | 261 |
| *IPS-1* | AGGTGGACATGATAACCC | AGGAAGATAAGACCGAGG | 224 |
| *MITA* | GCCAGCCTGTGGTTTAG | TGTTTCTGGTTACGACCTT | 105 |
| *TRAF2* | CGCATCAGCATCGTTTA | TGGATGCTGCCCTATAA | 151 |
| *TRAF3* | GTCAGTTCCACCGCTAC | TCCTCCACCTTGTTCTC | 133 |
| *TRAF6* | TGGATGGACGATTAGTG | CAAATGAAGATACCGTGA | 160 |
| *IKKγ* | CTTTCTAACGGTGGCTAA | CTCACAGCGTTGTCGTA | 286 |
| *IKKβ* | AGAGCAGTATGGAGGTA | TAGACTGTTCAAGTGGC | 297 |
| *TAK1* | TCTCAAGGGAGCAACGA | CAGAATGGTGCCGAATG | 267 |
| *IRF3* | TGTGATGGGCAGTTGGT | GGTTCGGTTAGACTGGG | 262 |
| *IRF7* | CGTGGGCAGAGGGCTAT | GGTGAAACAGGTGACGGGA | 214 |
| *p65* | TCAACCCAGTCCAAGCAGCA | GATGCTGCCAGCTGAACGTC | 107 |
| *IκBα* | TAGGCAGAACGGGTGTC | AGACGCCGAACTACGAT | 282 |
| *JNK* | TTCTTGTCAGCGAGCCA | CTGGGCTGACTTCACCT | 131 |
| *P38* | TGTTTCCCACAACTAATC | GGAATGAAGTGGCTCAA | 115 |
| *NOD1* | CTGTTCTGCTGGATTGT | ATCGTGAAGTCCCATACC | 244 |
| *NOD2* | GTGGGTACAGTGAACGA | GGAAGGTCCTGAAAGAG | 126 |
| *RIP2* | CTTCAGGGCAAGTGGCA | CAGGGTCGGATGCGTGA | 123 |
| *MyD88* | GCATCTTGCGCTTCCTCACC | CCTGGTCCTTGGTTACGGCA | 107 |
| *TLR1* | CCAGGTAGGTGAGGTGGCAG | GAGAGCCAGAAGGTGCTGCT | 175 |
| *TLR2* | TCTGCAAGCTGCGAAGAGTC | CCAGAACCTGGGAATCTGGC | 80 |
| *TLR3* | CTTCTCGTCTCGGCGGTGAT | GACCGCACTAAGGCTGAGGT | 135 |
| *TLR5* | GGTGGTAGGGAAGGTGGCTC | TGTCAGACTGTGCAGCGTCA | 200 |
| *TLR8* | CGTGGATGTGATCGTGCTGC | CTGTTCTGGGCCACTCCACA | 110 |
| *TLR9* | GCAGCGACTTCTGGACGAGA | CTCGGCCAGGACAACACAGA | 126 |
| *TLR13* | CCATCCCGACCATCACTCGT | CTCCCATCGGTGCATGCAAC | 132 |
| *TLR21* | GGACGTTCTCCTGCTCGTGT | AACAGCTCTTGGGCCTGTGT | 147 |
| *TLR1* | GGACCTTCAACTCCTCACTGACG | GTTGGGATGCTGCAGGAGATG | 94 |
| *PI3K* | GGCGTCTTGGAAACATC | CGATGCGGACGAGACAG | 113 |
| *AKT* | GAGGAGAGAGAGGAGTGGATGCG | CGCTGGGTGAGCCGAACAA | 106 |
| *IRAK4* | CAACGGCGGAGATAAAC | ACTGAGGTGGCGAATGT | 152 |
| *IRAK1* | CCGCTGTGGACACCTAC | CCAGACACTTGCACGCTA | 246 |
| *β*-actin | GGCTACTCCTTCACCACCACA | TCTCCAAGGCAACGGGTCT | 188 |
