# Supplementary material for: PRR-Mediated Immune Response and Intestinal Flora Profile in Soybean Meal-Induced Enteritis of Pearl Gentian Groupers, Epinephelus fuscoguttatus♀ × Epinephelus lanceolatus♂
Source: Front Immunol. 2022 Feb 28;13:814479. doi: 10.3389/fimmu.2022.814479 (PMC8919722; doi:10.3389/fimmu.2022.814479)
Supplement: Supplementary file 9 [file Table_9.docx]

**Supplementary Table 9** The envfit significance test of the intestinal flora OTU and key genes in RIG-like receptor signaling pathway (n=4)

| Genus | CCA1 | CCA2 | r2 | P |
| --- | --- | --- | --- | --- |
| Photobacterium | -0.8814 | 0.4724 | 0.8819 | 0.005 |
| Faecalibacterium | 0.9463 | -0.3232 | 0.902 | 0.002 |
| Stenotrophomonas | 0.8932 | -0.4497 | 0.839 | 0.005 |
| Vibrio | 0.9639 | -0.2661 | 0.8504 | 0.001 |
| Neisseria | 0.8816 | -0.4721 | 0.7996 | 0.005 |
| Bacteroides | 0.8602 | -0.51 | 0.8739 | 0.005 |
| unidentifiedRikenellaceae | 0.8751 | -0.4839 | 0.8334 | 0.005 |
| Streptococcus | 0.868 | -0.4966 | 0.8512 | 0.005 |
| Romboutsia | 0.8779 | -0.4789 | 0.824 | 0.005 |
| Subdoligranulum | 0.8683 | -0.4961 | 0.9065 | 0.002 |

**Supplementary Table 10** The envfit significance test of the intestinal flora OTU and key genes in NOD-like receptor signaling pathway (n=4)

| Genus | CCA1 | CCA2 | r2 | P |
| --- | --- | --- | --- | --- |
| Photobacterium | 0.8204 | -0.5717 | 0.9386 | 0.001 |
| Faecalibacterium | -0.7478 | 0.6639 | 0.9555 | 0.001 |
| Stenotrophomonas | -0.85 | 0.5267 | 0.9273 | 0.001 |
| Vibrio | -0.62 | 0.7846 | 0.891 | 0.003 |
| Neisseria | -0.8585 | 0.5127 | 0.9278 | 0.001 |
| Bacteroides | -0.8387 | 0.5446 | 0.9431 | 0.001 |
| unidentifiedRikenellaceae | -0.8435 | 0.5371 | 0.9201 | 0.001 |
| Streptococcus | -0.8282 | 0.5604 | 0.8867 | 0.002 |
| Romboutsia | -0.8368 | 0.5474 | 0.9135 | 0.002 |
| Subdoligranulum | -0.8368 | 0.5475 | 0.9565 | 0.001 |

**Supplementary Table 11** The envfit significance test of the intestinal flora OTU and key genes in TLR-like receptor signaling pathway (n=4)

| Genus | CCA1 | CCA2 | r2 | P |
| --- | --- | --- | --- | --- |
| Photobacterium | -0.9984 | -0.0562 | 0.9822 | 0.001 |
| Faecalibacterium | 0.9799 | 0.1996 | 0.9792 | 0.001 |
| Stenotrophomonas | 0.9994 | 0.0336 | 0.9602 | 0.001 |
| Vibrio | 0.9377 | 0.3474 | 0.9492 | 0.001 |
| Neisseria | 0.9997 | 0.0234 | 0.9446 | 0.001 |
| Bacteroides | 0.9998 | 0.0178 | 0.9809 | 0.001 |
| unidentifiedRikenellaceae | 0.9994 | 0.0359 | 0.9708 | 0.001 |
| Streptococcus | 0.9992 | 0.0396 | 0.9605 | 0.002 |
| Romboutsia | 0.9988 | 0.0481 | 0.9654 | 0.001 |
| Subdoligranulum | 0.9999 | 0.0163 | 0.9835 | 0.001 |
