# Supplementary material for: PRR-Mediated Immune Response and Intestinal Flora Profile in Soybean Meal-Induced Enteritis of Pearl Gentian Groupers, Epinephelus fuscoguttatus♀ × Epinephelus lanceolatus♂
Source: Front Immunol. 2022 Feb 28;13:814479. doi: 10.3389/fimmu.2022.814479 (PMC8919722; doi:10.3389/fimmu.2022.814479)
Supplement: Supplementary file 10 [file Table_12.docx]

**Supplementary Table 12** The envfit significance test of the key genes and inflammatory genes in RIG-like receptor signaling pathway (n=4)

| Gene | CCA1 | CCA2 | r2 | P |
| --- | --- | --- | --- | --- |
| LGP2 | 0.9076 | -0.4199 | 0.5779 | 0.026 |
| MDA5 | 0.9995 | -0.033 | 0.8964 | 0.001 |
| IPS1 | 0.9834 | -0.1814 | 0.9393 | 0.001 |
| TRAF2 | 0.946 | -0.3242 | 0.896 | 0.002 |
| TRAF3 | 0.9342 | -0.3567 | 0.94 | 0.001 |
| TRAF6 | 0.9095 | -0.4157 | 0.859 | 0.001 |
| TAK1 | 0.6888 | -0.725 | 0.778 | 0.005 |
| IRF3 | 1 | 0.0051 | 0.9732 | 0.001 |
| IRF7 | 0.9958 | -0.0918 | 0.9795 | 0.001 |
| IκBα | 0.9007 | -0.4345 | 0.7678 | 0.003 |
| p65 | 0.9899 | 0.1415 | 0.7743 | 0.003 |

**Supplementary Table 13** The envfit significance test of the key genes and inflammatory genes in NOD-like receptor signaling pathway (n=4)

| Gene | CCA1 | CCA2 | r2 | P |
| --- | --- | --- | --- | --- |
| NOD1 | 0.8573 | -0.5148 | 0.8448 | 0.004 |
| NOD2 | 0.9824 | 0.1868 | 0.9511 | 0.002 |
| RIP2 | 0.8703 | -0.4926 | 0.9202 | 0.001 |
| TAK1 | 0.6876 | -0.7261 | 0.9312 | 0.002 |
| JNK | 0.9689 | 0.2476 | 0.8395 | 0.001 |
| TRAF6 | 0.9195 | -0.3931 | 0.8854 | 0.001 |
| IκBα | 0.8921 | -0.4518 | 0.8347 | 0.001 |
| p65 | 0.9779 | 0.209 | 0.8026 | 0.004 |

**Supplementary Table 14** The envfit significance test of the key genes and inflammatory genes in TLR-like receptor signaling pathway (n=4)

| Gene | CCA1 | CCA2 | r2 | P |
| --- | --- | --- | --- | --- |
| TLR5 | 0.8232 | -0.5677 | 0.9255 | 0.001 |
| TLR8 | 0.8124 | -0.5831 | 0.9024 | 0.003 |
| TLR9 | 0.9583 | -0.2857 | 0.7407 | 0.003 |
| TLR21 | 0.9444 | -0.3289 | 0.8371 | 0.001 |
| TLR22 | 0.9994 | 0.0339 | 0.6942 | 0.01 |
| TAK1 | 0.6843 | -0.7292 | 0.9268 | 0.001 |
| IκBα | 0.8925 | -0.451 | 0.8313 | 0.001 |
| p65 | 0.9745 | 0.2243 | 0.808 | 0.004 |
| MyD88 | 0.9519 | 0.3065 | 0.9432 | 0.001 |
| AKT | 0.9935 | -0.1138 | 0.9559 | 0.001 |
| IRAK4 | 0.9711 | 0.2387 | 0.9408 | 0.001 |
